# Fetal-Maternal Interactions in the Synepitheliochorial Placenta Using the eGFP Cloned Cattle Model

**DOI:** 10.1371/journal.pone.0064399

**Published:** 2013-05-28

**Authors:** Flavia Thomaz Verechia Pereira, Lilian J. Oliveira, Rodrigo da Silva Nunes Barreto, Andrea Mess, Felipe Perecin, Fabiana Fernandes Bressan, Ligia Garcia Mesquita, Maria Angelica Miglino, José RodrigoValim Pimentel, Paulo Fantinato Neto, Flávio Vieira Meirelles

**Affiliations:** 1 University Estadual Paulista, Campus of Dracena, Dracena, Brazil; 2 Department of Veterinary Medicine, College of Animal Sciences and Food Engineering, University of São Paulo, Pirassununga, Brazil; 3 Department of Surgery - Anatomy of Domestic and Wild Animals - College of Veterinary Medicine and Animal Sciences - University of São Paulo, Butantã, Brazil; 4 Department of Animal Nutrition and Production - College of Veterinary Medicine and Animal Sciences - University of São Paulo, Butantã, Brazil; 5 Center for Cell-based Theraphy, College of Medicine of Ribeirao Preto, University of Sao Paulo, Ribeirao Preto, Brazil; University of California, San Diego, United States of America

## Abstract

**Background:**

To investigate mechanisms of fetal-maternal cell interactions in the bovine placenta, we developed a model of transgenic enhanced Green Fluorescent Protein (t-eGFP) expressing bovine embryos produced by nuclear transfer (NT) to assess the distribution of fetal-derived products in the bovine placenta. In addition, we searched for male specific DNA in the blood of females carrying *in vitro* produced male embryos. Our hypothesis is that the bovine placenta is more permeable to fetal-derived products than described elsewhere.

**Methodology/Principal Findings:**

Samples of placentomes, chorion, endometrium, maternal peripheral blood leukocytes and blood plasma were collected during early gestation and processed for nested-PCR for eGFP and testis-specific Y-encoded protein (TSPY), western blotting and immunohistochemistry for eGFP detection, as well as transmission electron microscopy to verify the level of interaction between maternal and fetal cells. TSPY and eGFP DNA were present in the blood of cows carrying male pregnancies at day 60 of pregnancy. Protein and mRNA of eGFP were observed in the trophoblast and uterine tissues. In the placentomes, the protein expression was weak in the syncytial regions, but intense in neighboring cells on both sides of the fetal-maternal interface. Ultrastructurally, our samples from t-eGFP expressing NT pregnancies showed to be normal, such as the presence of interdigitating structures between fetal and maternal cells. In addition, channels-like structures were present in the trophoblast cells**.**

**Conclusions/Significance:**

Data suggested that there is a delivery of fetal contents to the maternal system on both systemic and local levels that involved nuclear acids and proteins. It not clear the mechanisms involved in the transfer of fetal-derived molecules to the maternal system. This delivery may occur through nonclassical protein secretion; throughout transtrophoblastic-like channels and/or by apoptotic processes previously described. In conclusion, the bovine synepitheliochorial placenta displays an intimate fetal-maternal interaction, similar to other placental types for instance human and mouse.

## Introduction

Placental establishment in eutherian mammals requires interaction between the chorioallantoic membrane and the maternal uterine tissue [1]–[3]. Different degrees of invasiveness are observed among the different known types of placenta. The haemochorial placentae have invasive trophoblast that establish direct contact to the maternal blood [1], [2], [4]–[6]. This may lead to presence of fetal DNA or cells circulating on the maternal system [7]–[9]; this phenomenon is called fetal microchimerism [10]. In contrast, on epitheliochorial placentae, the uterine epithelium and the maternal blood vessels remain intact throughout gestation. Therefore, such placentae are regarded to have considerably less intense fetal-maternal contact than other placental types [1], [4]. Conversely, the bovine placenta is a more complex placental type, where different areas may possess different levels of fetal-maternal interaction. Furthermore, the arcade zones may represent specialized areas of cell-cell interaction between mother and fetus [11]–[15], which is characterized by the intense phagocytic and absorptivity activity of placental epithelium in ruminants [11]–[15]. For example, the trophoblast cells in the arcade zone express of major histocompatibility complex (MHC) proteins around four months of pregnancy whereas the villi trophoblast cells do not express MHC proteins [16]. Furthermore, specific fetal cells, the binucleated trophoblast giant cells (TGC), migrate towards the endometrial epithelium during a heterologous cell-fusion process [17], called the syncytial regions [11]–[13]. In the arcade zone, the distance between the trophoblast and maternal blood system is locally minimized [14]. In addition, there is some evidence in the bovine model that fetal DNA can reach the peripheral blood of the mother [15], [18]–[22]. Nevertheless, the ground laying mechanisms of this phenomenon are not well understood. To investigate the fetal-maternal cell interactions during the establishment of the synepitheliochorial placenta in bovine, we have developed a model of transgenic enhanced Green Fluorescent Protein (t-eGFP) expressing bovine embryos produced by nuclear transfer (NT) ([23], see [24], [25] for background information on cloning or NT).

Briefly, the exogenous gene eGFP was transduced by a lentiviral vector into bovine fibroblast cells previously isolated from a bull [25]. The transgene expressing fibroblast cells were then used as a nuclei donor to produce transgenic eGFP (t-eGFP) expressing bovine embryos by somatic cell nuclei transfer (NT) that were transferred into surrogate cows [23]. Due to the high solubility [26] and the absence of a signal peptide [27] in our eGFP construction, normal secretion of the gene products seems to be unlikely, thus, it is expected that the eGFP would be found in fetal cells only. However non-classical mechanisms of protein secretion may occur and delivery eGFP from fetal cells have to be considered [28]–[33]. Our model is unique to study the transfer of fetal components to the maternal system by the induction of expression of a transgene by the bovine placenta. Herein, we searched for male specific DNA in the maternal blood after transfer of in vitro produced (IVP) bovine embryos. Moreover, t-eGFP expressing bovine NT pregnancies were evaluated by molecular and microscopic techniques to assess placental distribution and the presence of the exogenous eGFP products in the maternal system. Our hypothesis is that the bovine placenta allows intimate fetal-maternal interaction comparable to that of other placental types.

## Materials and Methods

### Production by *In Vitro* Fertilization and NT of Pregnancies and Maternal Peripheral Blood Collection

Bovine embryos were produced by in vitro fertilization and transferred into synchronized, surrogate cows, following an established protocol [34]. Blood samples were collected from recipient cows on day 7 (before embryo transfer), day 39 (at the time pregnancy diagnosis by transrectal ultrasound) and day 62 (at the time of fetal sexing by transrectal ultrasound). Expression of a testis-specific Y-encoded protein (TSPY) was tested in the peripheral blood leukocytes (PBL, N = 30) and blood plasma (N = 32) at the day of embryo transfer to avoid detection of TSPY derived from previous pregnancies. Only cows that did not show any TSPY detectable expression at day 7 were included in this study. Samples from three cows carrying female embryos were included as negative control. For the eGFP model, 4 male t-eGFP expressing bovine NT pregnancies were produced [23]. The t-eGFP pregnancies were surgically interrupted at days 60 (N = 3) and 90 (N = 1) of gestation. Tissue and blood samples were collected from both dates. This research was approved by the Ethical Committee of the College of Veterinary Medicine and Animal Science of the University of Sao Paulo (protocol number: 1678/2009).

### Detection of TSPY Gene in the Maternal Blood

DNA from maternal peripheral blood leukocytes was extracted using salt precipitation method [18]. Cell-free fetal DNA of maternal blood was extracted using the QIAmp DNA mini kit (Qiagen, #51106; Valencia, CA, USA). Nested-PCR approach was used to amplify a fragment of the bovine TSPY DNA using the set of primers shown in Table 1 [18]. The first nested-PCR reaction contained 2.5 µL of 10x buffer solution, 0.75 µL (25 mM) of Mg_2_^+^Cl, 1.2 mM of each dNTP (Invitrogen, Carlsbad, CA, USA, #10297–018), 0.2 µM of each external primer (forward and reverse), 1.0 U of Taq Polimerase (Invitrogen, Carlsbad, CA, USA, #10342–053), template DNA and DEPC water to complete 25 µL total volume. Different amounts of template DNA were used to maternal peripheral blood leukocytes (100 ng/µL) and cell-free fetal DNA (5 µL, correspond to million maternal or 10 000 male genome–equivalent cells [35]). The DNA was amplified by an initial denaturation step of 94°C for 5 min, followed by 18 cycles of denaturation at 94°C for 45 s, annealing at 60°C by 45 s and elongation at 72°C for 45 s. An additional extension time of 10 min at 72°C was added at the end of the final cycle. Prepubertal female blood samples were used as negative control. For peripheral blood leukocytes or cell-free fetal DNA, the second PCR consisted of 10 µL SYBR Green® PCR Master Mix (Applied Biosystems, Foster City, CA, USA, #4309155), 0.2 µM of each internal primer (forward and reverse), 2 µL of the first peripheral blood leukocytes product and DEPC water to complete 20 µL. The DNA was amplified in Applied Biosystems 7500 Real-Time PCR Systems thermocycler (Applied Biosystems, Foster City, CA, USA) by an initial step of 50°C for 2 min and 95°C for 10 min; followed by 40 cycles of 95°C for 10 s and an additional step 60°C for 1 min. A dissociation curve was achieved with a temperature ramp from 60 to 95°C to check the specificity of the amplicon produced in each reaction. The PCR products were analyzed on 1.5% agarose gel and visualized by ethidium bromide staining. Only samples that were found to be expressed in the real-time PCR reactions and showed a single band (152 bp size) in the agarose gel were considered positive for TSPY expression.

**Table 1:** Primer/Probe sets used for qRT-PCR.

Gene Target	Primer/Probe	Sequence (5′–3′)	Annealing Temperature (°C)
Bovine testis-specific Y-encoded protein (TSPY) - External Primers	Forward^a^	CCCGCACCTTCCAAGTTGTG	60
	Reverse^b^	AGAAGACGGTGGAGGAGCA	
Bovine testis-specific Y-encoded protein (TSPY) - Internal Primers	Forward^a^	CATCGTGGAGGAGGTGGAGGTT	64
	Reverse^b^	TTGTCACCAGCAGTTGTCACG	
enhanced Green Fluorescent Protein (eGFP)	Forward^a^	CCACATGAAGCAGCACGACTT	60
	Probe	TTCAAGTCCGCCATGCCCGAA	
	Reverse^b^	TACGTCCAGGACCGCACC	
Bovine glyceraldehyde-3-phosphate dehydrogenase (GAPDH)	Forward^a^	AAGGCCATCACCATCTTCCA	60
	Probe	AGCGAGATCCTGCCAACATCAAGTGGT	
	Reverse^b^	CCACTACATACTCAGCACCAGCAT	

a Forward =  sense (5′) primer.

b Each probe was synthesized 6-FAM reporter dye and TAMRA quencher.

c Reverse =  antisense (3′) primer.

### Detection of eGFP Expression by Quantitative Real-Time PCR

Real time PCR was performed on samples from the chorion from the intercaruncular region (fetal tissue only), the intercaruncular endometrium (maternal tissue), as well placentomes (interdigitating maternal and fetal tissues). The mRNA was extracted using Allprep Kit (Qiagen, #80204, Valencia, CA, USA). RNA from the interplacentomal endometrium was extracted using TRIzol Reagent (Invitrogen, #15596–026 Grand Island, NY, USA) according to the manufacturer's instructions. Quantitative PCR reactions were performed according to [23]. Sequences of eGFP and endogenous control genes primers are described in Table 1. For the detection of eGFP by western blotting, 2 g of fetal chorion, placentomes and intercaruncular endometrium, from the pregnant horns, were used. The material was macerated and centrifuged. Then, quantification of the protein was performed by Braford colorimetric analysis and western blotting adapted from a previous protocol [36]. The protein homogenates were diluted in Tris-HCl buffer solution (TBS –10% of Sodium Dodecyl Sulfate (SDS); 20% of sucrose; 5% of β- mercaptoethanol; 125 mM, pH 6.8), and denatured at 100°C by 5 min, separated in 12% polyacrylamide gel in TBS, and transferred to a polyvinylidene fluoride (PVDF) membrane by electrophoresis. The membrane was incubated overnight in Tris-HCl buffer solution with Tween-20 (TBS-T, 10 mM Tris pH 7.6; 0.9% of sodium chloride; 0.3% of Tween-20) containing of 5% of fat-free milk. Primary antibody incubation was performed using anti-GFP (Living Colors GFP Monoclonal Antibody, Clontech, Mountain View, CA, USA, #632375) in a final concentration of 0.1 µg/ml in containing of 2% of fat-free milk (TBS-TB) for overnight at room tempeture; then washed and incubated for 90 min with Dako-advance HRP Link (DakoCytomation, Carpinteria, CA, USA, #K4069). The blots were developed using the ECL Plus Western blotting detection reagents (GE Healthcare, Piscataway, NJ, USA, # RPN2132). The membrane was exposed to X-ray film for 5 min.

### Immunolocalisation of eGFP in the Bovine Placenta

Placentomes from the cloned and normal pregnancies were fixed in 4% buffered paraformaldehyde, dehydrated in alcohol, diaphanized in xylene and paraffin embedded. For antigen recovering, silanized slices of 4–5 µm were deparaffinized, rehydrated and boiled in citrate buffer (1.83 mM of monohydrate citric acid and 8.9 mM of Sodium citrate tribasic dehydrate, pH 6.0) in a microwave for 20 minutes for antigen retrieval and cooled in room temperature for ∼25 min. After that, the sections were incubated in Tris buffered solution (TBS, 2.0 mM of Trizma base and 1.36 mM of sodium chloride, pH 7.5), supplemented with 3% hydrogen peroxide for 30 minutes in the dark. The sections were incubated in TBS containing 10% normal goat serum (Invitrogen, Carlsbad, CA, USA, #16210–0172) for 30 minutes to block the non-specific binding sites. Anti-GFP antibody(Living Colors GFP Monoclonal Antibody, Clontech, Mountain View, CA, USA, #632375) was incubated in concentration of 1 µg/ml and other section was incubated with an isotype control (mouse IgG1 isotype control, Sigma-Aldrich, Saint Luis, MS, USA, #M5284) diluted in 1% normal goat serum in TBS overnight at 4°C. Then, the sections were washed with 1% normal goat serum in TBS. The reaction was revealed using the kit Dako-advance HRP Link (DakoCytomation, #K4069), followed by incubation with 3,3′-diaminobenzidine (DAB Peroxidase Substrate Kit, Vector Laboratories, Burlingame, CA, USA, #SK-4100). The sections were counter-stained with Hematoxylin, dehydrated and cover-slip mounted for further light microscopy analysis.

### Ultrastructural Analysis by Transmission Electron Microscopy

To study the ultrastructure, samples of placentomes were perfusion-fixed with 2.5% glutaraldehyde, post-fixed in 1% osmium tetroxide, dehydrated in ethanol, treated with propylene oxide and embedded in Spurr’s epoxy resin (Electron Microscopy Sciences, Hatfield, PA, USA #RT14300). Semi thin sections, stained with toluidine blue, were examined by a light microscope. Ultrathin sections, stained with uranyl acetate and lead citrate, were analyzed by a Zeiss EM90 electron microscope (Carl Zeiss, Jena, Germany).

## Results

### Fetal DNA is Present in the Maternal Blood of the Pregnant Cow

TSPY DNA was present in the maternal peripheral blood leukocytes (PBL) and the cell-free fetal DNA in the plasma of cows carrying male IVF embryos. Detection of the TSPY gene showed an efficiency of 66.7% on day 39 (20/30) and 70.0% on day 62 (21/30) for peripheral blood leukocytes, and 43.8% (14/32) and 50% (16/32), respectively, for cell-free fetal DNA in the maternal blood plasma. On day 62 of gestation, when the embryos have been sexed, it was confirmed that the TSPY gene was found only in male embryos, whereas the control female embryos remained negative for TSPY.

Detection of eGFP DNA by real time PCR in samples from eGFP expressing pregnancies had 100% of efficiency on all samples [days 60 (N = 3) and 90 (N = 1)], whereas TSPY expression was detected only at day 60 (3/3), but not in the individual studied on day 90.

### eGFP is Expressed by Fetal and Maternal Tissues in the t-eGFP-derived Bovine Placenta

**Figure 1:**
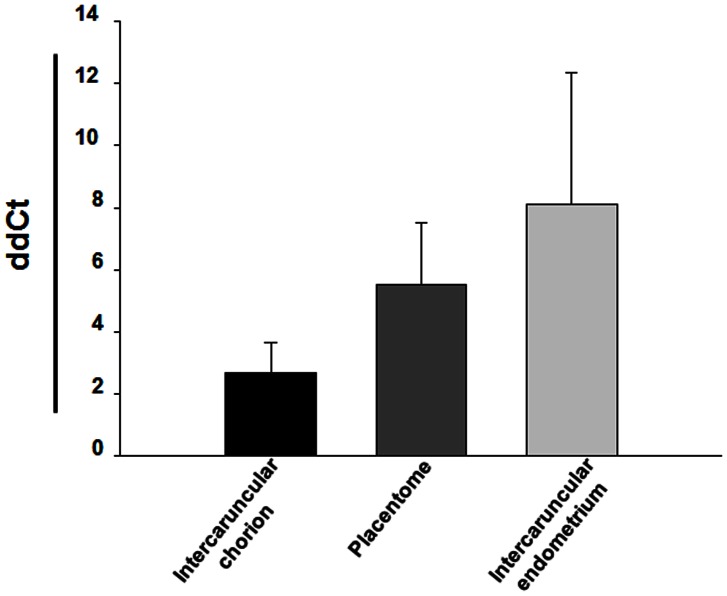
Expression of eGFP in fetal and maternal tissues from eGFP transgenic cloned placenta. Mean of relative expression of eGFP mRNA in the intercaruncular chorion (black bar), placentome (dark grey bar) and intercaruncular endometrium (light grey bar).

**Figure 2:**
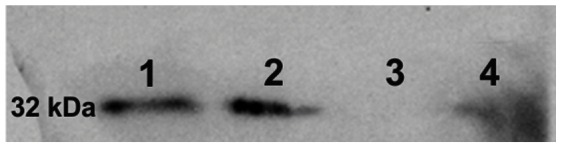
Detection of eGFP protein in fetal and maternal tissues from eGFP transgenic cloned placenta. Western blotting analysis to detect eGFP protein in 1: transgenic eGFP fetal chorion; 2: transgenic eGFP placentome; 3: molecular marker and 4: intercaruncular endometrium from pregnant horn eGFP from transgenic placenta.

The eGFP mRNA was present in the placentomes as well as in the chorion and the endometrium of the intercaruncular areas (Fig. 1). The chorion, as sample of fetal tissue only, showed the highest relative expression of eGFP mRNA compared to the placentome, which contained both maternal and fetal tissues. Interestingly, we found a low degree of eGFP expression in the intercaruncular endometrium (Fig. 1). Western blotting showed a specific band of 32 kDa, indicating the presence of the eGFP protein in the fetal tissues, such as the chorion, as well as in the placentomes. Moreover, eGFP was also detected in the intercaruncular endometrium of the pregnant horn, but not in that of the non-pregnant horn (Fig. 2).

### Immunolocalisation of the eGFP in the Bovine Placenta

**Figure 3:**
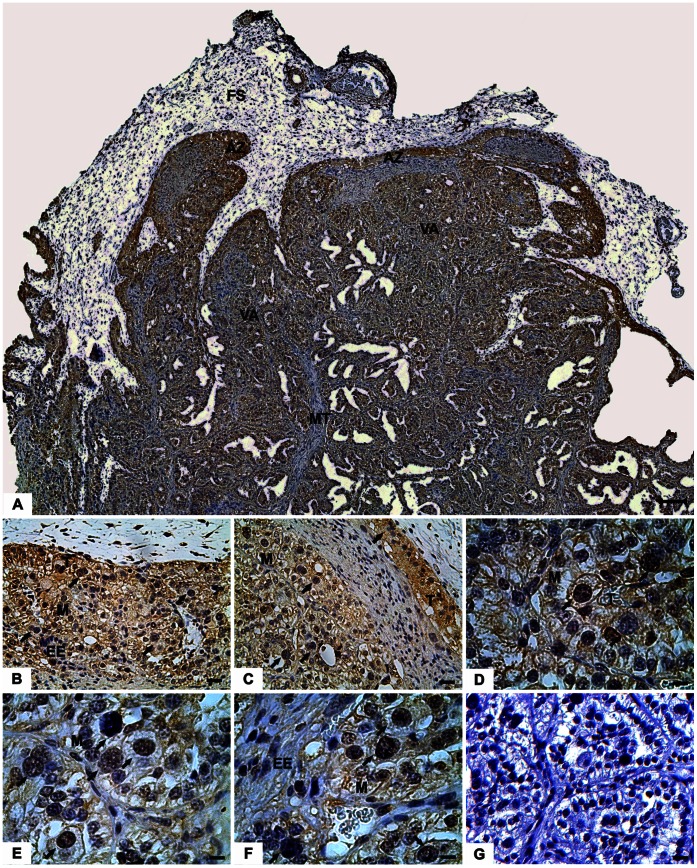
Expression of eGFP protein in the placentome at day 90. (A) On top the fetal side (FS) containing arcade zone (AZ) and villous areas (VA) with close intermingling of fetal and maternal tissues (MT). (B–C) Details of the arcade zone showing the trophoblast cells (T) and maternal uterine epithelium (M) positive for eGFP; endometrial stroma (EE) was mostly negative. Details of the arcade zone showed similar staining, including a binucleated trophoblast giant cell (arrows). Please note that some binucleate trophoblast giant cells are strong positive for eGFP while others are not. In (D–F), the presence of syncytial regions (arrows) in the villous region. Please note the fetal cells in close contact to the maternal epithelium displays weak staining to eGFP (arrows). (G) Negative control: placenta derived from a normal pregnancy that was stained for eGFP detection. trophoblast (T), endometrial stroma (EE), maternal uterine epithelium (M).

**Figure 4:**
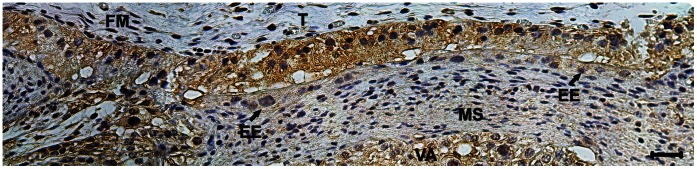
Enhanced green ﬂuorescent protein (eGFP) expression at the fetal-maternal interface of the eGFP transgenic cloned bovine embryo at day 90. Please note (arrow) that the trophoblastic cells that are close to the maternal cells displays weak staining for eGFP. Fetal mesenchyme (FM); endometrial epithelium (EE); maternal stroma (MS) and villous area (VA).

Immunohistochemistry analysis results showed the expression of the eGFP protein throughout trophoblast cells in the placentomal region (Fig. 3A–E). The figure 3A shows the expression of eGFP throughout the placentome, the eGFP is strongly stained in the fetal villi as compared to the maternal tree. Also, the mesenchyme showed to be highly positive for eGFP. In the placentome, the trophoblast cells of both the arcade zone (Fig. 3B–C) and the villous areas (Fig. 3D–F) showed positive staining for eGFP in all fetal cells including the most of binucleated giant trophoblast cells. Mesenchymal tissues were also positive for eGFP exhibiting lower expression of the exogenous protein (Fig. 3 A–F). Surprisingly, maternal uterine epithelium of the placentomes showed strong immunolabeling for eGFP, whereas the endometrial stroma was mostly negative (Fig. 3D–F). Likewise, the intercaruncular endometrium and isotype controls from NT pregnancies were negative (data not shown). As well, samples of placenta from normal pregnancies did not to be positive for eGFP (Fig. 3G). In the syncytial regions at the arcade zone (Fig. 4) and the villous area (Fig. 3E–F), the multinuclear fetal-maternal syncytia of TGCs and maternal epithelial cells showed a lower degree of protein expression (Fig. 4). In this region, the maternal vessels were located near the surface, and the uterine epithelium was more cuboid than columnar. In some areas, the epithelium was completely fused with the TGC (Fig. 3E–F).

### Fine Structure of the Fetal-Maternal Interface

**Figure 5:**
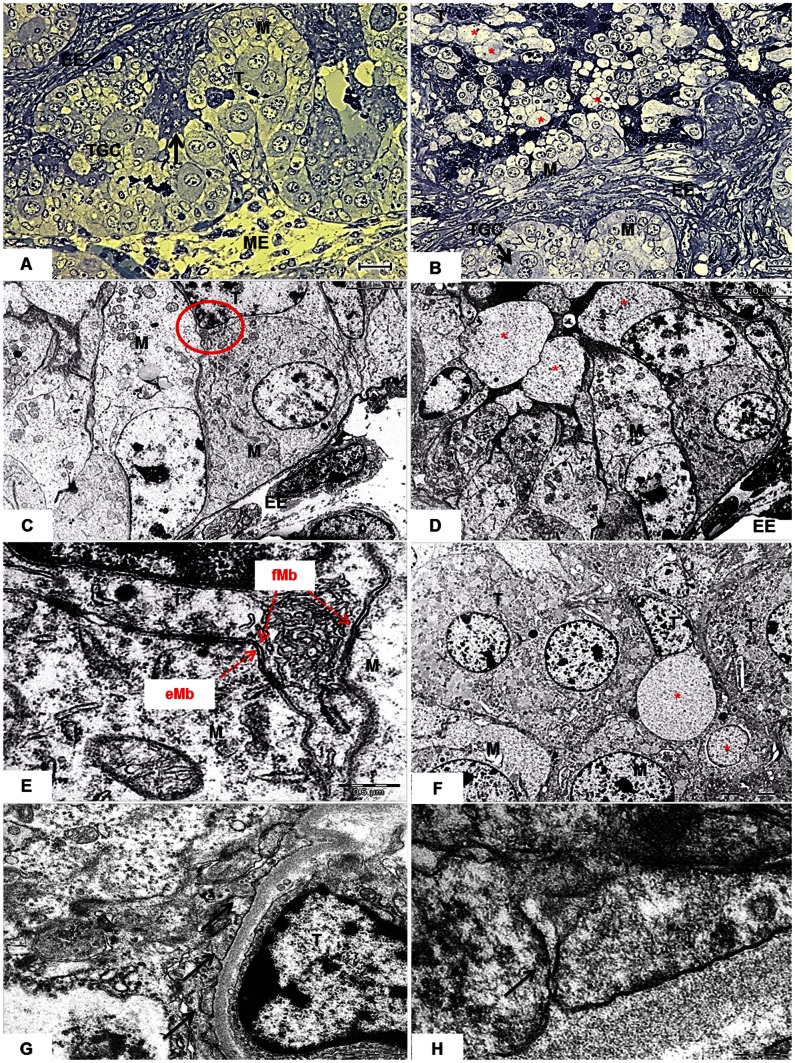
Semi-thin and ultrastructure of the fetal-maternal interface (day 90). (A–B) Light micrographs of semi-thin sections; (C–H) Transmission electron micrographs (TEM.) In A presence of syncytial region (arrow), endometrial stroma (EE), maternal epithelium (M), trophoblast (T), binucleate trophoblast giant cell (TGC) and mesenchyme (ME). In B, large vesicles (red asterisk) occurred in the interstitial spaces between trophoblast (T) and maternal epithelium (M). In C, TEM of a trophoblast cell (T) with the plasma membrane projection between two maternal epithelial cells (M) (red circle). In D, large vesicles (red asterisk ) close to maternal cells (M). In E, detail of the projection of membrane (Mb) of the trophoblast, please note the presence of desmosomes and epithelial (eMb) and trophoblastic (fMb) cell membranes are preserved (red arrows). In F, detail of trophoblast (T) cell with a large cytoplasmic vesicle (red asterisk) in close contact to maternal epithelium (M). In, G and H, the presence of channel-like channels (arrows) in the trophoblast (T) near the uterine epithelium.

Inside the placentomes, samples from obtained from NT pregnancies showed similar structural characteristics of the fetal-maternal interface that it was observed on the samples from in vivo pregnancies (Fig. 5). In the villous area, fetal and maternal systems were more closely apposed (Fig. 5A). Both fetal and maternal tissues displayed characteristics signs of apoptosis characterized by the enlargement of cellular limits and the presence of dense chromatin in both fetal and maternal cells (Fig. 5B). In the arcade zone, large interstitial gaps were observed between the trophoblast and the uterine epithelium containing large amount of vesicles (Fig. 5B). The maternal epithelial cell changed its morphology when is closely related to trophoblast cells from being columnar with a basal nuclei and large apical cytoplasm to cuboid-like cell with more centered nuclei with smaller apical cytoplasm area (Fig. 5C). Furthermore, there was a projection of the trophoblast cell membrane towards to the intercellular space of the uterine epithelial cells with the presence of desmosomes (Fig. 5C–E), suggesting that trophoblast cells can bypass the uterine epithelia barrier without disruption of its continuity. In parts, these cells were in intimate contact, besides there were a large number of vesicles that possibly is involved in the secretion of fetal factors to maternal cells (Fig. 5B, C, F). In some regions, there were trophoblasts cells that exhibited extremely large cytoplasmic vesicle which may result the accumulation of fetal secreted factors (Fig. 5F), these cells showed a blebbing formation out of the large granules that may represent the formation of smaller vesicles to be secreted to the maternal side (data not shown). Likewise to *in-vivo* produced pregnancies, TGCs and multinuclear syncytia of fetal and maternal cells were frequent (Fig. 3E–F). Furthermore, we found channel-like structures in the trophoblast plasma membrane near the uterine epithelium close to the maternal vessels. These structures were located on the apical surface, coursing deeply into the cytoplasm (Fig. 5E,F).

## Discussion

The synepitheliochorial placenta has been characterized as a type of placenta that has limited invasion of fetal cells towards to maternal side, since TGCs do not trespass the basal epithelial barrier [37]. However, it has been reported the presence of fetal male-specific fetal DNA circulating as early as day 30 of pregnancy in the cow [38]. In according to that, our results revealed the presence of cell-free fetal DNA for first time at days 39 and 62 of pregnancy in the cow. Furthermore, the presence of fetal DNA was also detected in the cellular compartment of maternal blood in both days 39 and 62 of bovine pregnancy. In fact, it is not known if the bovine placenta is permeable only for fetal cells products (i.e. nucleic acids and proteins) or if actually there is, in some level, the occurrence of cellular microchimerism during pregnancy in the cow as reported in other species [7]–[10]. Nevertheless, the mechanisms by how fetal DNA reaches the maternal circulation in the cow are still unclear.

In this context, we developed a transgenic eGFP (t-eGFP) expressing bovine embryos produced by somatic cell nuclear transfer to assess at placental and systemic levels the interaction between fetal and maternal system during the period of placentogenesis.

Indeed, our t-eGFP expressing pregnancy model showed to be a useful model to understand the putative mechanisms that might be involved in the bovine transplacental transport of fetal molecules and/or cells towards the maternal side.

The delivery of fetal contents such as nuclear acids (Fig. 1) and proteins (Fig. 2) through the placenta was detected in both local and systemic level. The eGFP protein was not only present in the fetal cells as expected, but surprisingly eGFP was present in the large majority of maternal epithelial cells (Fig. 3). We also have assessed the ultrastructure features of the t-eGFP expressing pregnancies to search for morphological evidences for the transplacental transport in the cow. It is known that clones may have alterations on the placental system [39], [40]; however the fine structure has not been investigated yet. The ultrastructure of our individuals from NT pregnancies however seemed to be similar to *in-vivo* produced embryos [11]–[15].

Firstly, we report the presence of fetal microchimerism for the TSPY gene (both cellular and cell-free fetal DNA) in IVF and eGFP expressing NT pregnancies during early gestation and it was efficient for pregnancy detection. In addition, the presence of exogenous eGFP-DNA was identified in the maternal circulation in our t-eGFP model. The detection of fetal specific molecules and exogenous transgenic DNA in the maternal blood confirmed our initial hypothesis that bovine placenta is somehow permeable to fetal contents during early pregnancy. Earlier reports showed that several genes, for instance, TSPY [18], [22], Y-chromosome-specific repeat sequences (Y-S4) [19], [20], sex-determining region Y (SRY) [21], [41], fetal mitochondrial DNA [15] and a transgene (casein beta 2/3) [19], [20] that can detected in the maternal peripheral blood leukocytes in the cow. In particular, the TSPY is most likely to be detected due to its multiple-copies in the bovine genome [42]. Altogether, our data indicate that fetal microchimerism may be a common feature during gestation in bovine as in humans (see [10]), despite the morphological differences between placental types.

Secondly, the presence of the eGFP was found locally in fetal tissues, such as the chorion, as well as in the fetal-maternal tissues of the placentomes at mRNA (Fig. 1) and protein level (Fig. 2). Moreover, eGFP mRNA and proteins were found at low levels in the intercaruncular endometrium of the pregnant horns (Figs. 1 and 2). In contrast, the non-pregnant horns did not show any eGFP expression. The intercaruncular region is more loosely attached to the smooth chorionic epithelium which result in a lesser intimate contact compared to the placentomal region [13]. Both areas were involved in the maintenance of fetal-maternal communication [43]. In particular, the cells of the intercaruncular endometrium are responsible for the uptake of histiotroph [44] and the modulation of the maternal immune system [45], which may explain the expression of fetal-derived products in the maternal tissue of both placentomes and interplacentome areas as reported here.

Thirdly, the immunohistochemistry analysis showed strong eGFP protein expression by the trophoblast cells. Moreover, the eGFP was detected in maternal epithelial cells of the placentome areas. The relatively weak staining of the syncytial regions may be caused by the existence of an ubiquitin promoter in our eGFP construct [23]: it is known from human placentas that most invasive cells do not express ubiquitin [46]. However, neighboring cells were strongly positive on both sides of the fetal-maternal interface in our t-eGFP model. Our data suggest that a transfer of fetal molecules perhaps occur especially in the placentomes, where an intense contact between fetal and maternal cells is established [13]. Possibly, the levels of eGFP in the intercaruncular endometrium were not strong enough to produce positive signals in the immunohistochemical analysis, but the presence of eGFP was detected by western blotting analysis.

Additionally, widely distribution of the eGFP in the maternal epithelia in the placentomal areas was unexpected and led us to analyze ultrastructurally the fetal-maternal interface. The presence of a large number of trophoblast-derived vesicles toward to maternal cells and the projections of trophoblast membrane in between maternal epithelial cells indicate that trophoblast cell may be capable to deliver their contents to the maternal side without breaking the maternal physical barrier. Furthermore, the presence of channel-like structures similar to previously described in the trophoblast cells close to the maternal epithelium and the associated vessels may be a way of transport of fetal molecules to the maternal side. Such channels have been previously described in ruminants [47] and other species [48]–[52], and are thought to transfer water and small soluble molecules.

Among the possibilities for eGFP delivery to the maternal side, the apoptosis and/or necrosis of fetal cells can be considered one potential mechanism since fetal cells release their contents close to the maternal epithelium. The occurrence of apoptosis in placental cells has been well documented in human [53]–[54]. In the cow, there are evidences that apoptosis is present during pregnancy [55]–[57]. The debris of apoptosis may be uptaken by cells of the luminal epithelium [58], which may explain the presence of the eGFP protein in the maternal cells within the placentomes. In humans, studies in vitro showed that in the presence of apoptotic bodies endothelial cells decrease their expression of ICAM1 and interleukin-6 (IL6) [58]. Furthermore, phagocytosis of the apoptotic bodies stimulates the downregulation of proinflammatory cytokines (IL1B, IL8, IL10 and CSF2) and the derivatives of arachidonic -acid metabolism (leukotriene C4 and tromboxane B2) and an increase of prostaglandin E2 (PGE2), and platelet-activating factor (PAF) reducing the local inflammation [59] in the pregnant uterus. Also, apoptosis is one mechanisms by which extravillous trophoblast are eliminated in the human placenta [53].

Furthermore, eGFP, which is an exogenous protein and does not possess a signal peptide, can be secreted from expressing cells in an unfolded or non-properly folded format via unconventional secretion pathways [60]. Cytoplasmic proteins which lack signal peptide sequence can be released to extracellular space by unconventional secretion pathway [32]. The unconventional secretion pathways can be affected by inﬂammation, starvation, or mechanical stress by cell–cell rearrangements during development and epithelial cell proliferation [32] which usually is present during placentogenesis. There are two main pathways involved in unconventional secretion of proteins: the *Caspase 1* or *Golgi Reassembly-Stacking Protein* (GRASP) pathways [61]. The expression of nod-like receptors (NLRs) in the human first trimester trophoblast suggests that the *Caspase-1* unconventional protein secretion in the placenta [62]–[63]. Regardless of unconventional secretion of cytoplasmic proteins that could explain eGFP leakage to the maternal system there are no evidences of these pathways are present in the bovine placenta.

In conclusion, the delivery of fetal contents to the maternal system was shown on systemic and local levels, and involved nuclear acids (TSPY and eGFP) and protein (eGFP). Our data suggest that there is transplacental delivery of fetal molecules to the maternal side and the mechanisms by which occur remains to be determined. Putative mechanisms for eGFP secretion may be the occurrence of the apoptosis at the fetal-maternal interface; the presence of unconventional secretion pathways such as *Caspase-1* pathway already described in human trophoblast cells and/or secretion of unfolded and/or misfolded eGFP from the fetal cells. Also, the presence of channel-like structure in the trophoblast cells that were already described in closely related species such as sheep may play a role on eGFP secretion to the maternal system. In conclusion, our data suggest that the synepitheliochorial bovine placenta possesses molecular and cellular relationship between fetal and maternal in regards of the exchange of their contents that are more intimate despite the placental morphology.
